# Identifying Who Benefits the Most from a Community Health Worker-Led Multicomponent Intervention for Hypertension

**DOI:** 10.1155/2024/6311938

**Published:** 2024-09-17

**Authors:** Meng Pan, Andrea Beratarrechea, Rosana Poggio, Hua He, Chung-Shiuan Chen, Jing Chen, Vilma Irazola, Adolfo Rubinstein, Jiang He, Katherine T. Mills

**Affiliations:** ^1^ Department of Epidemiology School of Public Health and Tropical Medicine Tulane University, New Orleans, LA, USA; ^2^ Institute for Clinical Effectiveness and Health Policy, Buenos Aires, Argentina; ^3^ Translational Sciences Institute Tulane University, New Orleans, LA, USA; ^4^ Department of Medicine Tulane University School of Medicine Tulane University, New Orleans, LA, USA

## Abstract

**Background:**

Uncontrolled hypertension is a major public health challenge in low- and middle-income countries. The Hypertension Control Program in Argentina (HCPIA) showed that a community health worker-led multicomponent intervention was effective for blood pressure (BP) reduction in resource-limited settings, but whether the intervention was equally effective across participant subgroups is unknown.

**Objective:**

To identify participants who benefit the most from the HCPIA BP control intervention.

**Methods:**

This secondary analysis used data from HCPIA, a successful 18-month cluster-randomized trial in 18 health centers with 1,432 low-income hypertensive patients in Argentina. Fifteen baseline characteristics were used to define subgroups. The proportion of controlled BP (<140/90 mmHg) was estimated using generalized linear mixed models with arm-by-subgroup interaction terms. The distribution of trial BP response among intervention patient subgroups was assessed.

**Results:**

Participants were 53.0% female, a mean age of 56 years, and 17.4% controlled BP at baseline. After the intervention, 72.9% of intervention and 52.2% of control participants had controlled BP. The intervention was more effective in physically inactive patients (OR = 2.76, 95% CI: 1.82 and 4.21; *p* for interaction = 0.04), moderately active patients (OR = 3.08, 95% CI: 1.90 and 4.99; *p* for interaction = 0.03), and those with uncontrolled BP at baseline (OR = 2.77, 95% CI: 2.15 and 3.57; *p* for interaction = 0.05). Among intervention participants, 20.2% had no BP response (BP change < −4 mmHg), 41.3% had a moderate BP response (BP change: −4 mmHg to −24 mmHg), and 38.5% had a high BP response (BP change > −24 mmHg). Women (*p*=0.01), those who were physically inactive (*p*=0.03), and those not taking antihypertensive medications at baseline (*p*=0.001) had the greatest BP response.

**Conclusion:**

The effect of the intervention was consistent across many subgroups with some key groups showing a particularly strong intervention effect. These findings could be useful for planning future hypertension control programs in low- and middle-income countries.

## 1. Introduction

Elevated blood pressure (BP) is a leading preventable risk factor for cardiovascular diseases (CVDs) and chronic kidney disease [1, 2]. The burden of hypertension is increasing globally, especially in low- and middle-income countries (LMICs) [3]. In 2010, an estimated 31.7% of men and 31.2% of women in LMICs had hypertension, including 30.4% of men and 32.7% of women in Latin America and the Caribbean. However, only 7.7% of hypertension patients in LMICs had their blood pressure controlled (defined as systolic/diastolic BP < 140/90 mmHg) [4]. Two systematic reviews of blood pressure reduction trials indicate that community health worker (CHW)-delivered interventions are effective for blood pressure reduction [5, 6]. A meta-analysis indicated that multilevel, multicomponent, and patient-based interventions are most effective and should be applied to improve hypertension control [7].

The Hypertension Control Program in Argentina (HCPIA) was an 18-month cluster-randomized trial conducted from April 2015 to October 2016 in low-income patients with hypertension comparing a CHW-led multicomponent intervention to usual care for BP control in Argentina [8]. The trial resulted in 22.1% (*p* < 0.001) greater hypertension control in the intervention group (72.9%) compared to the control group (52.2%) and a 4.8 mm·Hg (95% confidence interval (CI): 3.2 and 6.3) and 3.3 mm·Hg (95% CI: 2.3 and 4.3) greater reduction in systolic blood pressure (SBP) and diastolic blood pressure (DBP), respectively [8]. These results clearly demonstrate that the multicomponent intervention was effective for BP reduction and control. Since other BP control interventions have shown heterogeneity of effectiveness across patient characteristics, [9, 10] the next important question of interest is whether it was equally effective in all subgroups of participants or if some groups benefited more from the intervention. Understanding who benefits the most from this type of intervention will aid in the planning of future BP reduction interventions and programs. Therefore, the objective of this report is to examine the effectiveness of the intervention for BP control and the magnitude of BP response by subgroups defined by baseline characteristics [11].

## 2. Methods

### 2.1. Study Design and Setting

HCPIA was conducted within medical centers of the Remediar + Redes program in Argentina, a national public system for primary healthcare [12, 13]. The program provides free medications and healthcare to low-income patients without health insurance. Details of the study design and setting are described elsewhere, [13] and the study results have been published previously [8, 14]. In brief, 18 eligible clinics were randomized to either the intervention or enhanced usual care, and 1,432 patients with hypertension were recruited from the clinic patients. The differences in the proportion of BP control at 18 months between the intervention and control groups (primary outcome), as well as differences in net change in BP, medication adherence, and medication titration, were examined. The study was approved by the Tulane University and Hospital Italiano de Buenos Aires (Argentina) institutional review boards. All participants signed written informed consent prior to study participation.

### 2.2. Participants

Clinics were eligible for inclusion if they met previously published inclusion criteria, [13] including being affiliated with the Remediar + Redes program and employing CHWs in addition to general physicians and nurses. Eighteen medical centers representing diverse geography and stratified by geographic region were randomly assigned to either the multicomponent intervention group (*n* = 9) or the usual care group (*n* = 9).

Eligibility criteria for study participants were as follows: (1) aged 21 or older, (2) had hypertension (defined as SBP ≥140 mmHg and/or DBP ≥90 mmHg at 2 separate screening visits and/or use of antihypertensive medications), (3) lived with a spouse or another hypertension adult (≥21 years old), and (4) had a cell phone that could receive text messages [13]. The rationale for requiring participants to be living with a spouse or another person with hypertension was due to the household-based nature of the intervention and to enhance intervention compliance with social support and accountability. A total of 743 intervention and 689 control participants were recruited.

### 2.3. Intervention

The 18-month intervention consisted of monthly home visits from trained CHWs for the first six months and every two months after that. During the visits, participants received tailored instruction on home BP monitoring, medication adherence techniques (including receipt of 7-day pill boxes), and lifestyle modification (weight loss and maintenance, increasing physical activity, alcohol intake moderation, dietary sodium reduction, and eating a healthy diet, such as the dietary approaches to stop hypertension (DASH) diet). CHWs also assisted patients in goal setting, provided social support, and helped them schedule upcoming physician appointments. Primary care physicians in intervention clinics received education and training on standard treatment algorithms for stepped-care hypertension management and received audits and feedback on patients' BP levels [15]. Participants also received weekly tailored text messages focusing on lifestyle modifications and medication adherence.

Enrolled participants from control clinics received usual care for their blood pressure. CHWs in the control clinics continued their traditional roles of addressing maternal and child health and did not intervene with participants about their blood pressure.

### 2.4. Definitions and Measurements

Blood pressure was measured according to the American Heart Association recommendations using an automatic device (Intellisense Digital Blood Pressure Monitor; model: OMRON HEM-907 XL) with cuff sizes (pediatric, regular adult, large, or thigh) based on participant's arm circumference [16]. Participants were required to stay in a seated position after 5 minutes of quiet rest and to avoid alcohol, cigarettes, coffee/tea, and exercise for at least 30 minutes before their BP measurement. Trained and certified nurses obtained three BP measurements, and the average of the three was used for analysis. BP control was defined as SBP less than 140 mmHg and DBP less than 90 mmHg. BP response categories were defined based on the distribution of SBP change (termination SBP – baseline SBP) at 18 months and a previous meta-analysis as no response (SBP change ≥ −4 mmHg), moderate response (−24 mmHg < SBP change < −4 mmHg), and high response (SBP change ≤ −24 mmHg) [17].

A total of 15 baseline characteristics were used to define the subgroups of interest [9]. Binary variables included sex, current smoking (smoke ≥100 during lifetime and still smoking), alcohol drinking (drinks at least one day per week), high vegetable intake (consumes more than five servings of fruits and vegetables per day), adding salt to food (adds salt while preparing or consuming food most of the time or always), high risk of CVD (including history of myocardial infarction, stroke, diabetes, and hypercholesterolemia), baseline controlled BP (BP < 140/90 mmHg), and having any family member with hypertension. Age was classified as 21–49, 50–59, 60–69, and ≥70 based on the distribution. Body mass index (BMI) was divided into normal (<25 kg/m^2^), overweight (25–30 kg/m^2^), and obese (>30 kg/m^2^) groups. Weekly physical activity was quantified by the calculated metabolic equivalent of tasks (METs) as inactive (0 MET/week), insufficient (<12 MET/week), moderate (12–32 MET/week), and regular (>32 MET/week) based on previously used categories [18]. The number of antihypertension medications (assessed by medication inventory) was classified as 0, 1, and ≥2. Medical adherence was quantified using the 8-item Morisky Medication Adherence Scale and was classified as low (<6), medium (6≤ scores <8), and high (score = 8) medication adherence [19].

### 2.5. Statistical Analysis

Frequency and proportion are reported for categorical variables, mean and standard deviation for normally distributed continuous variables, and median and interquartile range (IQR) for non-normally distributed continuous variables. Data analysis was performed according to the intention-to-treat principle. Multilevel cluster effects were accounted for in all analyses using a compound symmetry covariance structure with family and clinic as random effects [20]. Generalized linear mix models (GLMMs) were used to estimate and compare differences in baseline characteristics between intervention and control groups. Generalized estimating equation (GEE) models with a logit link and binomial distribution for each subgroup were used to estimate the proportions of controlled BP in intervention and control arms. Odds ratios (ORs) were used to evaluate intervention effects, and interaction term *p* values (intervention ∗ subgroup) were used to test the intervention effectiveness by subgroup. In addition, GLMMs were used to estimate BP changes within the intervention group for each subgroup, and GEEs with a clogit link and multinomial distribution were used to assess the distributions of BP response levels by subgroup. Unadjusted results and results adjusted for important covariates, including baseline age, sex, history of CVD and hypercholesterolemia, alcohol drinking, physical activity, BMI, and SBP, are presented. These covariates were selected for adjustment to be consistent with adjustments in the main trial results [8]. A 2-sided *p* value of <0.05 was considered statistically significant. All analysis was performed using SAS 9.4 (SAS Institute Inc., Cary, NC).

## 3. Results

Eighteen clinics (9 in each group) with 743 individuals in the intervention group and 689 in the control group were included in these analyses. Baseline characteristics are presented in Table 1. The mean baseline age was 55.8 years, BMI was 31.6 kg/m^2^, waist circumference was 105.8 cm, and average physical activity time was 23.0 MET/week. About 53.0% of patients enrolled in the study were women, 33.8% did not exercise, 39.6% had hypercholesterolemia, 22.4% had diabetes, 31.7% drank alcohol weekly, and 19.2% were current smokers. At baseline, only 17.4% had controlled BP, and only 36.1% who took antihypertensive medications had high medication adherence. Overall, the baseline characteristics of patients were balanced between the intervention and control groups. However, the intervention group had a higher baseline vegetable intake, a higher proportion of use of added salt, and a greater proportion at high risk of CVD compared to the control group. The intervention group also had slightly higher baseline SBP and DBP, a higher proportion of antihypertensive medication intake, and more participants with a family member with hypertension.

At the end of 18 months, 72.9% of participants in the intervention group and 52.2% of patients in the control group had controlled BP (Table 2). The overall odds ratio (OR) for hypertension control in the intervention group compared to the control group was 2.45 (95% CI: 1.94 and 3.10). Intervention effects were consistent across subgroups of sex, age, BMI, smoking, drinking, vegetable intake, added salt, CVD risk, number of antihypertensive medications, medication adherence, and family members with hypertension. The intervention was more effective in baseline physically inactive patients (OR = 2.76, 95% CI: 1.82 and 4.21; *p* for interaction = 0.04) and those with moderate physical activity at baseline (OR = 3.08, 95% CI: 1.90 and 4.99; *p* for interaction = 0.03) compared to those with regular physical activity (OR = 1.43, 95% CI: 0.88 and 2.32). In addition, the intervention was effective in those with uncontrolled BP at baseline (OR = 2.77, 95% CI: 2.15 and 3.57; *p* for interaction = 0.05) but not in those with controlled BP at baseline (OR = 1.37, 95% CI: 0.71 and 2.63).

Table 3 shows BP changes across subgroups in intervention clinic participants over the 18-month intervention. The overall BP reduction in intervention clinic participants was 19.30 mmHg (95 CI: 17.90 and 20.78) and 12.20 mmHg (95% CI: 11.20 and 13.20) for SBP and DBP, respectively. Females had significantly greater BP reduction than males (*p*=0.01 for systolic and *p* < 0.01 for diastolic), and those with normal BMI had greater BP reduction than overweight and obese participants (*p*=0.02 for systolic and *p* < 0.01 for diastolic). In addition, those who were physically inactive at baseline had significantly greater BP reduction than those with more physical activity (*p*=0.02 for systolic and *p*=0.01 for diastolic). Furthemore, those with no baseline BP medications had a greater reduction in SBP than those on medication at baseline (*p*=0.03), and the oldest participants had more DBP reduction than other ages (*p* < 0.01). Across the 9 intervention centers, SBP change ranged from −28.83 mmHg (95% CI: −31.63 and −26.03) to −9.33 mmHg (95% CI: −13.43 and −5.22), and DBP change ranged from −18.29 mmHg (95% CI: −20.12 and −16.46) to −6.41 mmHg (95% CI: −9.23 and−3.60).

Results for categorical BP responses to the intervention by subgroups have a similar pattern to those observed for continuous BP change (Table 4). The distribution of participants into no response, moderate response, and high response was 20.2%, 41.3%, and 38.5%, respectively. Females responded better than males (*p*=0.01) with 41.5% of females compared to only 35.1% of males having a high response. Physically inactive participants had a greater response compared to those who exercise regularly (44.8% vs. 33.0%, *p*=0.03). Of those with controlled BP at baseline, 57.8% had no response to the intervention, compared to only 12.1% of those with uncontrolled BP at baseline. Similarly, those with no BP medications at baseline were more likely to have a moderate or high BP response compared to those taking antihypertensive medications at baseline.

## 4. Discussion

The HCPIA trial found that a CHW-led multicomponent intervention for BP control was effective in a primary care setting in Argentina serving low-income, uninsured patients [8]. These post hoc analyses extend the prior analysis by examining participant characteristics related to intervention effectiveness and the magnitude of BP change among subgroups. These analyses allow us to further explore the consistency of the intervention effects and to determine if there are groups that might benefit more than others from this type of intervention in the future. Overall, we found that the intervention effect is consistent across a wide range of subgroups and can, therefore, be used broadly in hypertensive patient populations to reduce BP and improve BP control. These findings, coupled with those published previously demonstrating the overall effectiveness and cost-effectiveness of the intervention, [8, 21, 22] suggest that it could be scaled-up in the healthcare system in Argentina and other LMICs for hypertension control. In addition, we have identified some groups that appear to benefit the most from the intervention, including those who are the most sedentary, women, those with uncontrolled BP, and those who are not taking antihypertensive medications prior to the intervention.

Studies of similar interventions have also reported consistent intervention effects among some predefined subgroups, [23, 24] and only a few analyzed outcomes by an extended number of baseline characteristics [9, 10]. Asche et al. tested a telemonitoring and pharmacist management intervention in hypertension patients in Minneapolis, and reported larger intervention effects in younger patients and in those with low salt intake, fewer antihypertensive medications, and uncontrolled DBP at baseline [9]. Consistent with our findings, Green et al. tested home blood pressure monitoring, web communication, and pharmacist care on hypertension control in Washington and Idaho and found that patients with higher baseline systolic BP experienced more of an intervention effect compared to patients with controlled BP.

We found that those with little or no physical activity at baseline were more likely to benefit from the intervention. Prior studies have indicated that CHW-led education has resulted in positive improvement in physical activity among Latino populations by facilitating and supporting patients' lifestyle change [25]. Previous studies suggest that lifestyle modifications, such as dietary changes and increasing exercise, were possible and are associated with CVD prevention [26].

Better hypertension control depends on improvements in care delivery, effective therapy, and increased medication adherence [27]. Our finding that females have a greater response to the intervention could be due to the higher awareness of high blood pressure and adherence to antihypertensive medications [28]. Exercise also contributes to BP reduction, [16] so those who were sedentary at baseline had the greatest room for improvement in increasing physical activity, leading to BP reduction. The finding that patients above age 70 years had greater reductions in DBP was likely due to their higher baseline DBP compared to younger patients [29]. Similarly, patients without previous antihypertensive treatment are more likely to have greater SBP reduction because initiation of treatment as part of the intervention will likely result in significant BP lowering.

Our finding that the intervention was consistently effective across a variety of subgroups could provide support for the implementation of home-based CHW-led multicomponent interventions in resource-limited settings given the high prevalence of these subgroups. For example, a review showed that the prevalence of hypertension in Argentina was 36.3% with only 20% blood pressure control in 2017 and that more than 65% of the population had little physical activity [30]. Given that the intervention was especially effective in those with uncontrolled BP and insufficient physical activity, scaling up the intervention program in Argentina would likely result in substantial BP reduction. Furthermore, while the HCPIA results demonstrate the effectiveness of this approach in the general population, our findings of specific groups that benefit the most from the intervention could be helpful for planning the implementation of blood pressure control interventions targeted at these subgroups.

This study has several strengths and limitations. First, due to cluster-randomization, some participant-level covariates are not balanced at baseline. Due to this, analyses have used multivariate adjustment for key variables to account for these imbalances. Second, the control group also experienced a modest reduction in blood pressure during the trial likely due to regression to the mean [31]. Third, the results presented here are a secondary analysis of a large trial that achieved a significant difference in blood pressure change between groups in an LMIC providing a unique opportunity to evaluate groups that may be best suited for BP reduction interventions. However, the trial was powered for the main effects, so there might not be sufficient power to detect significant differences by subgroup. These post hoc analyses are for hypothesis generation and exploratory analyses. Subgroup analyses from other trials testing similar intervention components in low-resource settings should be conducted to shed light on the findings reported here.

Overall, the intervention appears to be consistently effective across a wide range of subgroups, suggesting it could be broadly effective in primary care settings in low- and middle-income countries. Furthermore, some groups seem to respond particularly well to this invention, including women, those who are physically inactive, and those with uncontrolled BP and not on antihypertensive medications. Due to the potential for great benefit from this intervention in those groups, multicomponent interventions could be targeted to these groups in future hypertension control programs to maximize the effectiveness and cost-effectiveness of intervention delivery in low-resource settings in low- and middle-income countries.

## Figures and Tables

**Table 1:** Baseline characteristics of the HCPIA study participants by randomization group.

Characteristic	Intervention (*N* = 743)^∗^	Control (*N* = 689)^∗^	*p* value
Female, no. (%)	394 (52.6)	378 (53.4)	0.5
Age, mean (SD), years	56.1 (13.6)	55.5 (13.0)	0.5
21–49, no. (%)	205 (28.4)	211 (29.8)	0.5
50–59	252 (31.9)	206 (32.1)	
60–69	197 (28.4)	203 (27.5)	
70+	89 (11.4)	69 (10.7)	
BMI, mean (SD), kg/m^2^	31.8 (6.6)	31.5 (6.5)	0.4
Normal, no. (%)	81 (10.7)	75 (11.2)	0.6
Overweight	243 (33.1)	235 (33.8)	
Obese	418 (56.2)	377 (55.0)	
Waist, mean (SD), cm	105.6 (14.5)	106.0 (14.7)	0.6
Physical activity, mean (SD), MET/week	21.8 (44.1)	24.2 (59.7)	0.4
Inactive, no. (%)	267 (35.1)	215 (32.4)	0.3
Insufficient	171 (25.0)	187 (25.0)	
Moderate	154 (20.4)	142 (21.2)	
Regular	147 (20.5)	145 (21.5)	
Current smoker, no. (%)	144 (19.2)	134 (19.2)	1.0
Weekly alcohol drinking, no. (%)	247 (33.4)	208 (30.1)	0.2
High vegetable intake, no. (%)	41 (5.4)	13 (2.0)	<0.01
Added salt, no. (%)	395 (53.9)	297 (43.4)	<0.01
High risk of CVD, no. (%)	416 (56.3)	341 (49.5)	0.01
Major CVD	93 (12.7)	62 (9.0)	0.03
Hypercholesterolemia	313 (42.4)	254 (36.8)	0.04
Diabetes	175 (23.7)	114 (21.1)	0.3
SBP, mean (SD), mmHg	151.7 (16.8)	149.8 (15.5)	0.03
DBP, mean (SD), mmHg	92.2 (12.2)	90.1 (12.9)	<0.01
Controlled hypertension, no. (%)	127 (17.0)	122 (17.6)	0.8
Number of antihypertensive medications			
0	104 (14.2)	114 (17.5)	0.03
1	439 (60.2)	415 (62.3)	
≥2	178 (24.6)	133 (20.2)	
Morisky score, median (IQR)	6.3 (4.4, 8.2)	6.3 (4.3, 8.3)	0.7
Low adherence, no. (%)	158 (26.9)	137 (24.0)	0.2
Medium adherence	256 (40.6)	217 (38.0)	
High adherence	206 (34.5)	217 (38.0)	
Family members with hypertension, no. (%)	522 (53.9)	429 (44.7)	<0.01

SD, standard deviation; BMI, body mass index; MET, metabolic equivalent; CVD, cardiovascular disease; SBP, systolic blood pressure; DBP, diastolic blood pressure; IQR, interquartile range. ^∗^Model-predicted proportions were reported from a generalized linear mix model with a random intercept for family and clinic.

**Table 2:** Proportion of controlled blood pressure at 18 months and intervention effects between intervention and control groups overall and by subgroups.

Characteristic	The proportion of controlled BP in the intervention group (*N* = 709)^a^ %	The proportion of controlled BP in the control group (*N* = 648)^a^ %	Intervention effects, OR (95% CI)	Interaction *P*^b^
Overall	72.9	52.2	2.45 (1.94, 3.10)	—
Sex				
Male	68.9	51.1	2.12 (1.53, 2.95)	0.2
Female	76.3	53.1	2.83 (2.06, 3.90)	
Age				
21–49	74.8	57.0	2.23 (1.46, 3.41)	0.3
50–59	69.3	49.3	2.32 (1.56, 3.44)	0.3
60–69	72.1	49.6	2.62 (1.68, 4.10)	0.5
70+	80.8	54.0	3.59 (1.65, 7.82)	
BMI				
Normal	76.2	58.3	2.29 (1.12, 4.68)	1.0
Overweight	77.8	53.7	3.03 (2.01, 4.56)	0.3
Obese	69.3	49.9	2.26 (1.67, 3.06)	
Baseline physical activity per week				
Inactive	78.8	57.4	2.76 (1.82, 4.21)	0.04
Insufficient	69.2	46.3	2.60 (1.67, 4.05)	0.07
Moderate	72.0	45.5	3.08 (1.90, 4.99)	0.03
Regular	67.7	59.5	1.43 (0.88, 2.32)	
Current smoker				
No	73.7	52.0	2.59 (1.99, 3.35)	0.4
Yes	69.5	53.3	1.99 (1.20, 3.29)	
Weekly alcohol drinking				
No	74.6	51.4	2.77 (2.08, 3.68)	0.1
Yes	69.5	54.1	1.94 (1.30, 2.89)	
High vegetable intake				
No	72.9	52.2	2.46 (1.94, 3.13)	0.6
Yes	75.7	45.6	3.72 (0.95, 14.60)	
Added salt				
No	74.0	54.6	1.18 (2.37, 1.71)	0.6
Yes	72.0	49.0	1.32 (2.67, 1.90)	
Risk of CVD				
No major CVD	73.4	51.7	2.58 (2.02, 3.30)	0.2
Major CVD	68.8	58.8	1.55 (0.78, 3.06)	
Hypercholesterolemia				
No	73.7	50.2	2.77 (2.05, 3.74)	0.2
Yes	71.8	55.6	2.03 (1.40, 2.95)	
Diabetes				
No	73.8	53.0	2.50 (1.91, 3.26)	0.8
Yes	69.6	49.4	2.34 (1.45, 3.80)	
Baseline BP control				
No	71.0	46.9	2.77 (2.15, 3.57)	0.05
Yes	81.9	76.8	1.37 (0.71, 2.63)	
Baseline medication intake				
0	78.9	67.4	1.81 (0.96, 3.41)	0.7
1	74.0	49.3	2.93 (2.15, 3.99)	0.2
≥2	69.1	49.5	2.28 (1.34, 3.88)	
Medical adherence				
Low adherence	63.3	45.0	2.10 (1.30, 3.39)	0.2
Medium adherence	72.0	45.3	3.11 (2.09, 4.64)	1.0
High adherence	79.1	54.8	3.11 (1.99, 4.86)	
Family member with hypertension				
No	72.6	47.9	2.88 (1.94, 4.26)	0.3
Yes	73.0	55.0	2.21 (1.65, 2.96)	

^a^Model-predicted proportions are reported. ^b^Interaction *p* values from generalized estimating equation models with a logit link and binomial distribution. Each of the 15 subgroup analysis models includes one subgroup predictor, the treatment group, and one interaction of the treatment group by subgroup variable. The *p* value is for the treatment group by subgroup interaction.

**Table 3:** Blood pressure changes over 18-month intervention in 709 intervention clinic participants.

Characteristic	*N*	Crude mean SBP change (95% CI), mmHg	Adjusted mean SBP change (95% CI) ^∗^, mmHg	Adjusted P	Crude mean DBP change (95% CI), mmHg	Adjusted mean DBP change (95% CI) ^∗^, mmHg	Adjusted *P*
Overall	709	−19.30 (−20.78, −17.90)	−18.71 (−20.75, −16.67)	—	−12.20 (−13.20, −11.20)	−13.24 (−14.67, −11.80)	—
Sex							
Male	329	−17.52 (−19.49, −15.55)	−17.30 (−19.45, −15.15)	0.01	−11.33 (−12.64, −10.03)	−12.22 (−13.71, −10.72)	<0.01
Female	380	−20.28 (−22.21, −18.36)	−20.12 (−22.58, −17.65)		−13.14 (−14.51, −11.77)	−14.25 (−15.99, −12.52)	
Age							
21–49	199	−19.80 (−22.31, −17.30)	−19.22 (−21.80, −16.64)	0.8	−14.36 (−16.27, −12.44)	−11.12 (−13.01, −9.24)	<0.01
50–59	243	−17.40 (−19.89, −14.91)	−17.80 (−20.69, −14.91)		−11.25 (−12.99, −9.50)	−11.67 (−13.58, −9.76)	
60–69	187	−20.43 (−23.23, −17.63)	−18.99 (−21.51, −16.47)		−11.83 (−13.44, −10.22)	−13.88 (−15.57, −12.19)	
70+	80	−18.46 (−22.26, −14.66)	−18.83 (−21.95, −15.71)		−11.34 (−14.37, −8.30)	−16.27 (−18.58, −13.96)	
BMI							
Normal	74	−23.48 (−26.84, −20.12)	−20.96 (−24.04, −17.87)	0.02	−14.99 (−17.67, −12.30)	−14.65 (−17.02, −12.29)	<0.01
Overweight	234	−17.96 (−20.35, −15.58)	−18.38 (−20.99, −15.76)		−12.79 (−14.55, −11.03)	−13.75 (−15.52, −11.98)	
Obese	400	−18.77 (−20.72, −16.81)	−16.80 (−18.89, −14.71)		−11.51 (−12.77, −10.24)	−11.31 (−12.78, −9.83)	
Baseline physical activity							
Inactive	250	−22.24 (−24.60, −19.88)	−21.52 (−24.16, −18.89)	0.02	−14.43 (−16.11, −12.74)	−15.13 (−16.85, −13.40)	0.01
Insufficient	165	−18.61 (−21.23, −15.99)	−17.93 (−20.40, −15.47)		−12.16 (−13.91, −10.41)	−13.24 (−15.03, −11.44)	
Moderate	149	−16.11 (−18.92, −13.31)	−17.83 (−20.47, −15.19)		−10.79 (−12.84, −8.73)	−12.39 (−14.35, −10.43)	
Regular	145	−16.86 (−20.15, −13.57)	−17.55 (−20.79, −14.31)		−10.38 (−12.44, −8.32)	−12.19 (−14.36, −10.03)	
Current smoker							
No	573	−19.01 (−20.62, −17.39)	−18.73 (−20.86, −16.59)	1.0	−12.10 (−13.21, −10.99)	−12.98 (−14.48, −11.49)	0.2
Yes	136	−18.98 (−22.32, −15.65)	−18.65 (−21.66, −15.63)		−13.17 (−15.41, −10.94)	−14.14 (−16.25, −12.03)	
Weekly alcohol drinking							
No	476	−19.63 (−21.41, −17.84)	−19.32 (−21.44, −17.19)	0.3	−12.10 (−13.28, −10.91)	−13.17 (−14.75, −11.59)	0.9
Yes	231	−17.73 (−20.09, −15.37)	−18.10 (−20.67, −15.53)		−12.64 (−14.36, −10.93)	−13.30 (−15.02, −11.58)	
High vegetable intake							
No	667	−19.28 (−20.80, −17.77)	−18.83 (−20.82, −16.84)	0.4	−12.42 (−13.47, −11.37)	−13.34 (−14.76, −11.92)	0.2
Yes	40	−14.52 (−21.15, −7.89)	−16.59 (−22.76, −10.41)		−9.48 (−13.19, −5.76)	−11.00 (−14.63, −7.37)	
Added salt							
No	329	−19.54 (−21.74, −17.35)	−19.28 (−21.50, −17.07)	0.3	−12.47 (−13.88, −11.06)	−13.73 (−15.24, −12.22)	0.3
Yes	378	−18.56 (−20.38, −16.74)	−18.21 (−20.67, −15.74)		−12.22 (−13.58, −10.86)	−12.88 (−14.60, −11.16)	
Risk of CVD							
No major CVD	622	−19.08 (−20.60, −17.55)	−19.36 (−20.70, −18.02)	0.5	−12.34 (−13.42, −11.26)	−13.24 (−14.20, −12.27)	1.0
Major CVD	87	−18.48 (−23.07, −13.88)	−18.06 (−21.75, −14.37)		−12.04 (−14.85, −9.22)	−13.23 (−15.75, −10.72)	
Hypercholesterolemia							
No	411	−18.84 (−20.64, −17.04)	−18.73 (−20.90, −16.57)	1.0	−12.43 (−13.73, −11.13)	−13.50 (−15.05, −11.94)	0.5
Yes	298	−19.22 (−21.44, −17.00)	−18.69 (−21.15, −16.22)		−12.13 (−13.57, −10.70)	−12.98 (−14.67, −11.28)	
Diabetes							
No	545	−18.88 (−20.55, −17.20)	−19.02 (−21.24, −16.81)	0.4	−12.43 (−13.61, −11.26)	−13.27 (−14.77, −11.77)	0.9
Yes	163	−19.47 (−22.35, −16.58)	−17.89 (−20.58, −15.19)		−11.89 (−13.82, −9.96)	−13.21 (−15.14, −11.28)	
Baseline BP control							
No	590	−22.46 (−23.92, −20.99)	−19.24 (−21.33, −17.16)	0.1	−14.48 (−15.52, −13.43)	−13.40 (−14.86, −11.94)	0.3
Yes	119	−1.45 (−3.87, 0.96)	−16.05 (−20.09, −12.00)		−1.29 (−3.14, 0.56)	−12.28 (−14.69, −9.88)	
Number of antihypertensive medications at baseline							
0	98	−20.42 (−23.45, −17.39)	−21.92 (−25.42, −18.43)	0.03	−14.28 (−16.56, −12.00)	−14.14 (−16.52, −11.75)	0.3
1	422	−19.02 (−20.79, −17.25)	−18.78 (−21.06, −16.51)		−12.52 (−13.75, −11.28)	−13.47 (−15.05, −11.89)	
2	137	−20.22 (−23.55, −16.89)	−18.01 (−20.88, −15.14)		−12.29 (−14.43, −10.15)	−12.95 (−15.04, −10.86)	
≥3	32	−15.21 (−22.54, −7.89)	−12.03 (−18.23, −5.84)		−7.06 (−11.25, −2.88)	−10.35 (−13.98, −6.72)	
Medical adherence							
Low adherence	154	−19.29 (−22.39, −16.19)	−16.69 (−19.80, −13.58)	0.2	−11.39 (−13.54, −9.24)	−11.88 (−14.03, −9.74)	<0.01
Medium adherence	246	−18.52 (−21.06, −15.98)	−19.36 (−21.94, −16.77)		−11.41 (−13.13, −9.70)	−12.47 (−14.27, −10.67)	
High adherence	197	−19.18 (−21.88, −16.48)	−19.00 (−21.51, −16.50)		−13.27 (−15.18, −11.36)	−14.24 (−16.04, −12.44)	
Family member with hypertension							
No	215	−21.32 (−23.85, −18.78)	−18.93 (−21.44, −16.41)	0.8	−12.99 (−14.65, −11.34)	−12.68 (−14.36, −11.00)	0.3
Yes	494	−17.78 (−19.56, −16.01)	−18.58 (−20.86, −16.30)		−11.94 (−13.21, −10.68)	−13.55 (−15.18, −11.93)	
Clinic district							
Buenos Aires (BA), Berisso	86	−25.20 (−27.27, −23.30)	−25.15 (−27.10, −23.21)	<0.01	−18.26 (−20.10, −16.46)	−18.29 (−20.12, −16.46)	<0.01
BA, Lomas de Zamora	79	−19.61 (−23.34, −15.89)	−19.61 (−23.34, −15.87)		−11.09 (−13.31, −9.05)	−12.19 (−13.34, −9.05)	
Entre Rios	79	−18.34 (−20.51, −16.34)	−18.37 (−20.49, −16.25)		−15.34 (−16.89, −12.46)	−15.57 (−17.14, −13.99)	
Marcos Paz	78	−18.14 (−22.17, −13.87)	−18.11 (−22.26, −13.95)		−13.65 (−16.21, −11.54)	−13.93 (−16.31, −11.56)	
Misiones north	77	−9.20 (−13.63, −5.28)	−9.33 (−13.43, −5.22)		−5.81 (−8.22, −3.54)	−6.41 (−9.23, −3.60)	
Misiones south	88	−20.24 (−21.78, −18.69)	−20.38 (−21.98, −18.79)		−12.07 (−13.49, −10.66)	−12.07 (−13.49, −10.66)	
Tucumán 1	73	−18.29 (−21.67, −15.03)	−18.31 (−21.57, −15.04)		−11.08 (−13.63, −9.77)	−12.18 (−14.53, −9.82)	
Tucumán 2	79	−28.33 (−31.65, −26.18)	−28.83 (−31.63, −26.03)		−13.53 (−14.27, −11.46)	−13.14 (−14.91, −11.37)	
Corrientes	70	−10.24 (−13.29, −6.81)	−10.21 (−13.59, −6.82)		−6.28 (−8.87, −3.88)	−6.36 (−8.98, −3.74)	

^∗^Generalized linear mix models were used and adjusted for baseline age, sex, history of CVD and hypercholesterolemia, alcohol drinking, physical activity, BMI, and SBP (or DBP).

**Table 4:** Categorical blood pressure response to the 18-month intervention in 709 intervention clinic participants by subgroup.

Characteristic	No response (>−4 mmHg), *N* (%)	Moderate response (−4 to −24 mmHg), *N* (%)	High response (<−24 mmHg), *N* (%)	Crude OR (95% CI)	*p* value	Adjusted OR (95% CI)^∗^	*p* value
Sex							
Female	71 (18.2)	150 (40.4)	159 (41.5)	1.31 (1.00, 1.71)	0.05	1.47 (1.08, 2.00)	0.01
Male	72 (22.5)	143 (42.4)	114 (35.1)				
Age							
21–49	33 (19.1)	90 (40.9)	76 (40.1)	1.02 (0.63, 1.65)	0.9	0.99 (0.59, 1.66)	1.0
50–59	60 (22.3)	92 (42.2)	91 (35.5)	0.84 (0.52, 1.35)	0.5	0.89 (0.53, 1.51)	0.7
60–69	34 (19.0)	79 (40.8)	74 (40.2)	1.03 (0.63, 1.67)	0.9	0.90 (0.54, 1.52)	0.7
70+	16 (19.4)	32 (41.0)	32 (39.6)	1		1	
BMI							
Normal	6 (11.5)	29 (34.1)	39 (54.4)	2.05 (1.25, 3.37)	<0.01	1.80 (0.91, 3.57)	0.09
Overweight	48 (21.6)	104 (42.4)	82 (36.0)	0.97 (0.71, 1.30)	0.8	1.07 (0.69, 1.65)	0.8
Obese	89 (21.0)	160 (42.2)	151 (36.8)	1		1	
Baseline physical activity per week							
Inactive	37 (16.0)	103 (39.1)	110 (44.8)	1.65 (1.14, 2.38)	<0.01	1.56 (1.04, 2.36)	0.03
Insufficient	38 (20.0)	60 (41.7)	67 (38.3)	1.26 (0.84, 1.90)	0.3	1.07 (0.68, 1.68)	0.8
Moderate	33 (23.6)	68 (43.0)	48 (33.5)	1.02 (0.67, 1.56)	0.9	1.02 (0.65, 1.59)	0.9
Regular	35 (24.0)	62 (43.0)	48 (33.0)	1		1	
Current smoker							
No	116 (20.4)	239 (41.4)	218 (38.2)	0.93 (0.63, 1.35)	0.7	0.97 (0.65, 1.46)	0.9
Yes	27 (19.2)	54 (40.8)	55 (40.0)				
Weekly alcohol drinking							
No	90 (19.2)	198 (41.1)	188 (39.7)	1.18 (0.88, 1.58)	0.3	1.15 (0.83, 1.61)	0.4
Yes	52 (21.9)	95 (42.2)	84 (35.9)				
High vegetable intake							
No	133 (20.0)	274 (41.1)	260 (39.0)	1.33 (0.74, 2.39)	0.3	0.98 (0.51, 1.89)	1.0
Yes	10 (24.9)	17 (42.7)	13 (32.4)				
Added salt							
No	63 (18.6)	130 (40.4)	136 (41.0)	1.20 (0.91, 1.58)	0.2	1.36 (1.01, 1.83)	0.05
Yes	80 (21.6)	161 (41.8)	137 (36.6)				
Risk of CVD							
No CVD	122 (20.2)	263 (41.3)	237 (38.5)	1.01 (0.65, 1.56)	1.0	1.03 (0.65, 1.64)	0.9
Major CVD	21 (20.3)	30 (41.4)	36 (38.4)				
Baseline BP control							
No	77 (12.1)	243 (42.8)	270 (45.2)	9.97 (6.79, 14.65)	<0.01	1.95 (1.14, 3.33)	0.01
Yes	66 (57.8)	50 (34.6)	3 (7.6)				
Baseline medication intake							
0	13 (15.5)	42 (39.1)	43 (45.3)	1.53 (0.97, 2.43)	0.07	2.50 (1.43, 4.39)	<0.01
1	84 (19.5)	174 (41.9)	164 (38.6)	1.16 (0.83, 1.62)	0.4	1.45 (0.99, 2.11)	0.06
≥2	38 (22.1)	71 (42.8)	60 (35.1)	1		1	
Medical adherence							
Low	28 (21.2)	72 (41.4)	54 (37.4)	0.87 (0.58, 1.31)	0.5	0.66 (0.42, 1.04)	0.07
Medium	56 (22.4)	101 (41.8)	89 (35.8)	0.81 (0.56, 1.18)	0.3	0.83 (0.57, 1.22)	0.4
High	41 (18.9)	73 (40.3)	83 (40.8)	1		1	
Family member with hypertension							
No	39 (19.0)	91 (40.8)	85 (40.2)	1.11 (0.82, 1.50)	0.5	0.77 (0.54, 1.08)	0.1
Yes	104 (20.7)	202 (41.6)	188 (37.8)				

^∗^Generalized estimating equation models with a clogit link and multinomial distribution were used and adjusted for baseline age, sex, history of CVD and hypercholesterolemia, alcohol drinking, physical activity, BMI, and SBP.

## Data Availability

The data used to support the findings of this study are available from the corresponding author upon reasonable request.
